# Identification of Networks of Co-Occurring, Tumor-Related DNA Copy Number Changes Using a Genome-Wide Scoring Approach

**DOI:** 10.1371/journal.pcbi.1000631

**Published:** 2010-01-01

**Authors:** Christiaan Klijn, Jan Bot, David J. Adams, Marcel Reinders, Lodewyk Wessels, Jos Jonkers

**Affiliations:** 1 Division of Molecular Biology, The Netherlands Cancer Institute, Amsterdam, The Netherlands; 2 Information and Communication Theory Group, Delft University of Technology, Delft, The Netherlands; 3 Netherlands Bioinfomatics Centre, Nijmegen, The Netherlands; 4 Experimental Cancer Genetics, Wellcome Trust Sanger Institute, Wellcome Trust Genome Campus, Hinxton, Cambridge, United Kingdom; University of Washington, United States of America

## Abstract

Tumorigenesis is a multi-step process in which normal cells transform into malignant tumors following the accumulation of genetic mutations that enable them to evade the growth control checkpoints that would normally suppress their growth or result in apoptosis. It is therefore important to identify those combinations of mutations that collaborate in cancer development and progression. DNA copy number alterations (CNAs) are one of the ways in which cancer genes are deregulated in tumor cells. We hypothesized that synergistic interactions between cancer genes might be identified by looking for regions of co-occurring gain and/or loss. To this end we developed a scoring framework to separate truly co-occurring aberrations from passenger mutations and dominant single signals present in the data. The resulting regions of high co-occurrence can be investigated for between-region functional interactions. Analysis of high-resolution DNA copy number data from a panel of 95 hematological tumor cell lines correctly identified co-occurring recombinations at the T-cell receptor and immunoglobulin loci in T- and B-cell malignancies, respectively, showing that we can recover truly co-occurring genomic alterations. In addition, our analysis revealed networks of co-occurring genomic losses and gains that are enriched for cancer genes. These networks are also highly enriched for functional relationships between genes. We further examine sub-networks of these networks, core networks, which contain many known cancer genes. The core network for co-occurring DNA losses we find seems to be independent of the canonical cancer genes within the network. Our findings suggest that large-scale, low-intensity copy number alterations may be an important feature of cancer development or maintenance by affecting gene dosage of a large interconnected network of functionally related genes.

## Author Summary

It is generally accepted that a normal cell has to acquire multiple mutations in order to become a malignant tumor cell. Considerable effort has been invested in finding single genes involved in tumor initiation and progression, but relatively little is known about the constellations of cancer genes that effectively collaborate in oncogenesis. In this study we focus on the identification of co-occurring DNA copy number alterations (i.e., gains and losses of pieces of DNA) in a series of tumor samples. We describe an analysis method to identify DNA copy number mutations that specifically occur together by examining every possible pair of positions on the genome. We analyze a dataset of hematopoietic tumor cell lines, in which we define a network of specific DNA copy number mutations. The regions in this network contain several well-studied cancer related genes. Upon further investigation we find that the regions of DNA copy number alteration also contain large networks of functionally related genes that have not previously been linked to cancer formation. This might illuminate a novel role for these recurrent DNA copy number mutations in hematopoietic malignancies.

## Introduction

Tumor development is generally thought to be a process in which healthy cells transform into malignant tumor cells through the step-wise acquisition of oncogenic alterations [1],[2]. This implies that certain changes have to occur together for effective oncogenic transformation of a normal cell. There are a multitude of (epi-)genetic lesions that cause deregulated expression of oncogenes and tumor suppressor genes. Co-operative deregulation of cancer genes has indeed been observed in several different settings. Retroviral insertional mutagenesis screens in mice have shown preferential co-mutation of specific combinations of genes within the same tumor [3]. Likewise, in a study where a thousand individual tumors were screened for mutations in 17 different oncogenes, preferential co-mutation of the *PIK3CA* and *KRAS* genes was observed [4].

Besides single basepair mutations or retroviral integrations, the activity of genes can also be perturbed by DNA copy number alterations that arise as a result of genomic instability, which is frequently observed in tumor cells [1]. Whether genomic instability is important for tumor initiation is controversial, but its contribution to tumor progression is undisputed [5],[6]. Loss of DNA is a mechanism for the tumor to eliminate copies of tumor suppressor genes, which prevent cancer formation. Conversely, DNA copy number gain or amplification may lead to activation of oncogenes that promote tumor development. We aimed to find genomic regions of gains and losses that are preferentially gained or lost together. We could subsequently link genes that lie in co-occurring regions to each other, allowing us to find functional interactions that reveal the mechanisms underlying tumor development.

DNA copy number alterations (CNAs) may be measured on microarray platforms [7]. Array-based comparative genomic hybridization (aCGH) of differentially labeled tumor and normal (2*n*) DNA is performed on oligonucleotide- or Bacterial Artificial Chromosome (BAC) based microarray platforms. For each probe on the microarray, the ratio of signal intensities of tumor versus normal DNA is a measure of the relative DNA copy number of the corresponding genomic region in the tumor sample. Platforms designed to identify single nucleotide polymorphisms (SNPs) can also infer CNAs by comparing the raw probe intensity values measured in a tumor sample with a reference sample.

**Figure 1:**
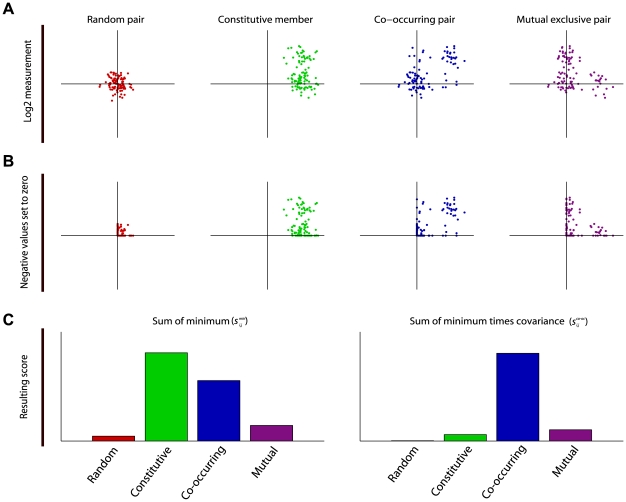
Co-occurrence score for paired continuous variables. a. Four possibilities of pairs of hypothetical DNA copy number change measurements are shown, for a set of samples. Each of the four hypothetical measurement pairs is plotted in scatter plot, giving each sample in the set an x- and y-coordinate. The random pair (first panel) is a noisy pair containing no effect. The constitutive member pair (second panel) consists of one measurement that is continuously high, paired with a measurement that varies between two noisy levels. The co-occurring signal (third panel) consists of two noisy measurements that alternate between a high and a basal level, but show concerted change. The mutual exclusive pair (fourth panel) also alternates between two levels but one measurement excludes the other from also reporting a high value. b. In this example we show scoring for co-occurring gains. Therefore we set all negative values to zero. To score for loss-loss pairs we would need to set all positive value to zero and continue using the absolute values. For loss-gain analysis we would set the positive values of the x (y) axis to zero and use the absolute values in the x (y) direction. c. The first panel shows the resulting scores of the four pairs of measurements if only the sum of the minimum is used. The second panel shows the score when the covariance is included.

**Figure 2:**
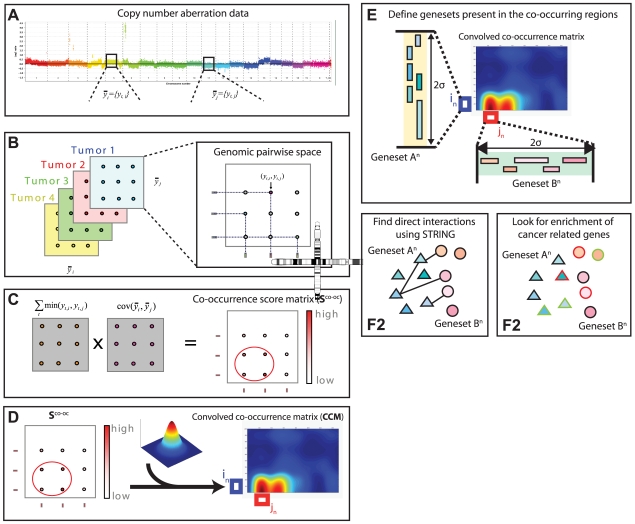
Schematic overview of co-occurrence analysis. a. Overview of aCGH data. Both 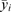 and 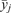 are vectors of genomic grid points spanning a chromosome arm (see Materials and Methods). The genomic grid is constructed from aCGH probe measurements, as explained in the Materials and Methods section. b. The combinations of 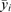 and 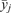 are used to construct a genomic pair-wise space in which all further calculations are performed. In this panel a schematic view of the genomic pair-wise space is shown. Each pair of genomic grid points between 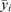 and 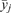 is a point in this space and each point contains two values. A pair-wise genomic matrix exists for each tumor in the data set. c. To score for co-occurrence, the minimum value of the pairs of genomic grid points are summed over the tumors and the co-variance over tumors of all genomic grid points is calculated. This results in two equally sized matrices which are multiplied element wise to produce the co-occurrence score matrix. This matrix is again represented in the genomic pair-wise space (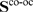). d. The co-occurrence score matrix is convolved with a Gaussian matrix to find local enrichment of high co-occurrence scores in the pair-wise space. Peaks in the convolved co-occurrence matrix are translated back to two genomic regions (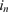 and 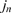) that are annotated as being co-aberrated across the tumor set. e. For the *n*-th peak in the Convolved Co-occurrence Matrix (CCM) two gene sets, 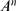 and 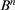, are defined, based on a 2σ window centered on the peak. f1. Using a protein-protein interaction database the interactions between gene sets derived from a single co-occurrence peak are analyzed, producing a set of interactions (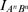). f2. Using the Cancer Gene Census we inspect the resulting gene sets for presence of known tumor-suppressor genes and oncogenes.

In order to extract those DNA copy number aberrations that preferentially occur together, we developed an analysis framework. The basic premise of our analysis is to define a pair-wise score for any given pair of genomic locations present in the dataset. This scoring index will only be high if both genomic locations are recurrently aberrated in multiple independent samples within the tumor panel, and if they co-vary similarly over the different samples (Figure 1). Using a Gaussian kernel convolution method we look for aggregates of high scores in the 2D genomic pair-wise space (Figure 2). The top peaks in the convolved score matrix can be mapped back to two distinct co-mutated genomic locations. The genes that reside in these genomic locations can then be functionally related to each other.

The raw data consist of non-discrete measurements of the average DNA copy number of the population of cells present in the measured sample. The signal consists of a measurement of a heterogeneous population of tumor cells, which may contain many populations potentially carrying different mutations and copy number alterations, as well as normal (diploid) cells. To reduce heterogeneity as much as possible we choose to analyze a collection of hematopoietic tumor cell lines, which on a per-sample basis can be considered clonal. There were several other reasons for analyzing this particular dataset. First, it is a high resolution dataset of well-characterized, clinically relevant samples. Although these samples are cell lines, they are widely used as a model system for the diseases from which they have been derived. Second, this collection of samples includes cell lines derived from T- and B-cell leukemias carrying rearranged T-cell receptor and immunoglobulin loci, respectively. We therefore should be able to separate these two distinct lymphoid malignancies based on co-occurring DNA copy number losses at the T-cell receptor and immunoglobulin loci. During T- and B-cell development, these loci undergo DNA recombination and gene deletion in a process known as V(D)J-recombination. The human genome contains three specific T-cell receptor loci (alpha/delta, beta and gamma) on two different chromosomes that determine their variability. B-cells have three different loci (IgG kappa, IgG lambda and the IgG heavy chain) on three different chromosomes that undergo recombination to generate a diverse repertoire of immunoglobulins. Since T- and B-cells only undergo recombination of their respective loci after lineage commitment, it is unlikely that T-cell receptor loci are recombined in B-cells and vice-versa. If our approach is successful at finding co-occurring losses, it should identify the co-occurring rearrangements at the T-cell receptor alpha/delta and beta/gamma loci in T-cell leukemias. Similarly, we should be able to pick up co-occurring losses at the IgG kappa, lambda and heavy chain loci in B-cell malignancies.

## Results

### Defining a continuous co-occurrence score

A classic example of finding associations in a large (binary) dataset is association rule mining. Identification of cooperating events in continuous data requires a different approach than binary association rule mining. First we developed a method to score for co-occurrence between two continuous measurements (Figure 1). We then applied this score in a framework that is able to find co-occurrences in genome-wide measurements. This framework is shown in Figure 2 and is detailed in the Materials and Methods.

DNA copy number measurements at two different genomic loci can be visualized in a 2D space, with each axis representing measurements at a certain genomic locus. A point in this space represents a sample in which both loci were measured. Figure 1a shows four hypothetical combinations of measurements. We sought to score for co-occurring high or low values in the DNA copy number data; in other words, regions that display similar patterns of large-amplitude amplification and deletion across the tumor set. This situation is shown in the third panel Figure 1a. The other panels show other potential situations that may arise when comparing two continuous measurements. To score for co-occurring gains all negative values are set to zero (Figure 1b). To score for co-occurring losses all positive values need to be set to zero and the absolute values of the measurements need to be used.

We use the covariance of the two measurements to score for co-occurring loci. This score only rewards a high value to a truly co-occurring and co-varying pairs of measurements (Figure 1c, right panel). However, a high covariance alone is not sufficient, since it is possible that a high covariance occurs while at least one of the loci never reaches a high amplitude (see Figures 1e and 1f).For this reason we multiply the covariance score with the sum of the individual valued in each sample. This method of scoring only rewards a high value to a co-varying pair of measurements with a large aberration amplitude across the tumors (Figure 1c, right panel).

### A framework for genome-wide co-occurrence scoring

The co-occurrence scores can be computed for every pair of genomic loci (Figure 2c). By performing a two-dimensional Gaussian kernel convolution on these scores in the co-occurrence space we can take local neighborhood effects into account. This operation is performed for different kernel widths in order to capture scale dependent effects, resulting in a Convolved Co-occurrence Matrix (CCM) as shown in Figure 2d. High values in this matrix represent candidate co-occurring regions in the data. A peak in the CCM can be mapped back to two specific loci, the size of which is determined by the *σ* parameter of the Gaussian function used to convolve the score matrix (Figure 2e). The genes that are located in the loci associated with a peak in the CCM are subsequently investigated. We examined both enrichment for known cancer genes in these gene lists and we investigated functional relationships between the genes derived from the two loci (Figure 2f). Additional details can be found in the Materials and Methods section.

### Co-occurrences in hematological cell lines

We ran our analyses on the aCGH profiles of 95 hematological tumor cell lines analyzed on the Affymetrix Genome-Wide Human SNP Array 6.0. See the supplemental data (Dataset S1) for a list of the cell lines that were analyzed. The data was generated by the Cancer Genome Project (Wellcome Trust Sanger Institute, Hinxton, UK). We employed three scale parameters: 2Mb (σ = 1/3 Mb), 10Mb (σ = 5/3 Mb) and 20Mb (σ = 10/3 Mb). In the remainder of this text we will refer to these as Scales 2, 10 and 20. These scales roughly determine the size of the aberrant regions we expect to find. By employing a small, medium and large scale we maximize the chance of detecting co-occurring changes of all possible sizes. To remain conservative we limited our primary analysis to the top 50 peaks in the Convolved Co-occurrence Matrix (CCM) for each of the scales and each of the comparisons (gain-gain, loss-loss, loss-gain). This resulted in 9 top-50 lists of co-occurring regions retrieved from this dataset.

### Co-occurrences involving the T-cell receptor and IgG loci

**Figure 3:**
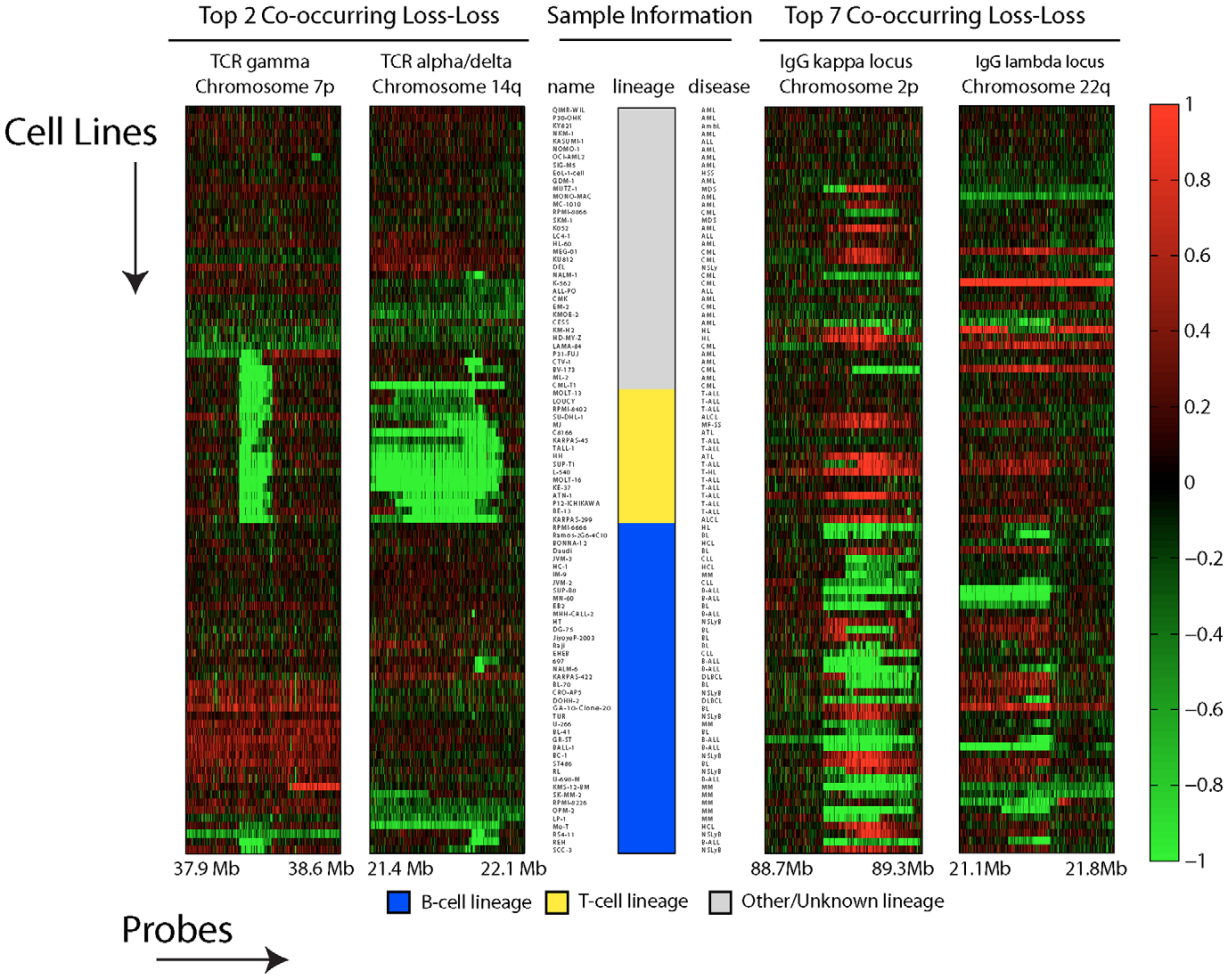
Two co-occurring losses detected in the 2Mb scale analysis. Raw aCGH data of two co-occurring losses corresponding to four genomic loci are shown. The y-axis of the heatmaps contains the samples, ordered through standard hierarchical clustering. The x-axis contains the probes present in the four genomic loci, ordered by genomic location. The sample information bar contains the names of the cell lines analyzed, the disease of origin and the whether the sample has a T-cell or B-cell lineage. These representations are based on the results of the analysis on the 2 Mb scale.

A substantial fraction of the 95 cell lines are derived from T- or B-cell lymphomas with functionally rearranged T-cell receptor or IgG genes. We therefore expected to identify co-occurring losses at the T-cell receptor alpha/delta and beta/gamma loci in the T-cell leukemias. Similarly, our method should identify co-occurring losses at the IgG kappa, lambda and heavy chain loci in B-cell malignancies. Because the recombination loci for both the T-cell receptor and the IgG genes are both relatively small (in the 1Mb range) we expected to retrieve these co-occurring losses in the small (2 Mb) scale analyses. Since we disregarded co-occurrences on the same chromosome we expected to find five co-occurring losses. Indeed, four of the five expected co-occurring losses are present in the top 50 peaks of the Scale 2 analyses (Table 1). Figure 3 shows two examples from the top 50 lists of co-occurring loci. The separation of T- and B-cell lines is immediately apparent. T-cell lines are strongly associated with losses in the T-cell receptor loci. A large subset of B-cell lines are associated with losses in the IgG loci. However, a subset of the B-cell lines is not associated with any loss of these loci. In this particular subset of lines the IgG loci seem to be gained. It is known that the IgG loci are favorite partners for oncogenic translocations [8]. Whether this is the cause of the amplification of these loci is not known.

**Table 1:** Occurrence of T-cell and B-cell related co-occurring losses.

Cell lineage	Loci of interaction	Rank in interaction list (scale 2)
**B-cell**	IgG kappa – IgG lambda	7
	IgG kappa – IgG heavy chain	6
	IgG lambda – IgG heavy chain	3950
**T-cell**	TCR beta – TCR alpha/delta	1
	TCR gamma – TCR alpha/delta	2

### Cancer gene enrichment in co-occurring loci

While the recovery of the V(D)J-related recombination loci as co-occurring losses serves as a positive control for our analysis approach, we are mainly interested in identifying cooperating genes or regions that might play a role in cancer. To see whether the locations we recover are linked to this disease, we analyzed whether the co-occurring genomic loci are enriched for genes known to play a role in cancer. As a reference gene set we used the Cancer Gene Census list [9]. The results of this analysis are shown in Table 2. As can be seen, the co-lost loci are mainly enriched for tumor suppressor genes, and the gain-gain regions for oncogenes. Since one expects loss of tumor suppressors and gain of oncogenes, this is a logical result, further increasing our confidence that our approach identifies truly relevant genomic loci.

**Table 2:** Enrichment for Cancer Gene Census genes in top 50 co-occurring genomic loci.

Comparison	Analysis Scale (Mb)	Genes present in co-occurring loci	CGC^2^ – Oncogenes (n – pvalue^1^)	CGC^2^ – Suppressor (n – pvalue^1^)
**Loss-loss**	2	198	2 - 0.56901	2 - **0.03481**
	10	1151	18 - 0.35721	7 - 0.0546
	20	1912	31 - 0.27088	11 - **0.039637**
**Gain-Gain**	2	221	10 - ***0.00050472***	0 - 1
	10	755	17 - **0.034575**	2 - 0.51641
	20	1192	28 - ***0.0065355***	4 - 0.43198
**Gain-Loss**	2	86	2 - 0.1373	2 - ***0.0037163***
	10	785	15 - 0.12887	4 - 0.15322
	20	1479	31 - **0.021555**	5 - 0.44561

1 p-value determined by Fishers' Exact test, p<0.05 is marked in bold script, p<0.01 is marked in bold italic script.

2 Cancer Gene Census.

### Discovering functional relationships between co-occurring loci

**Figure 4:**
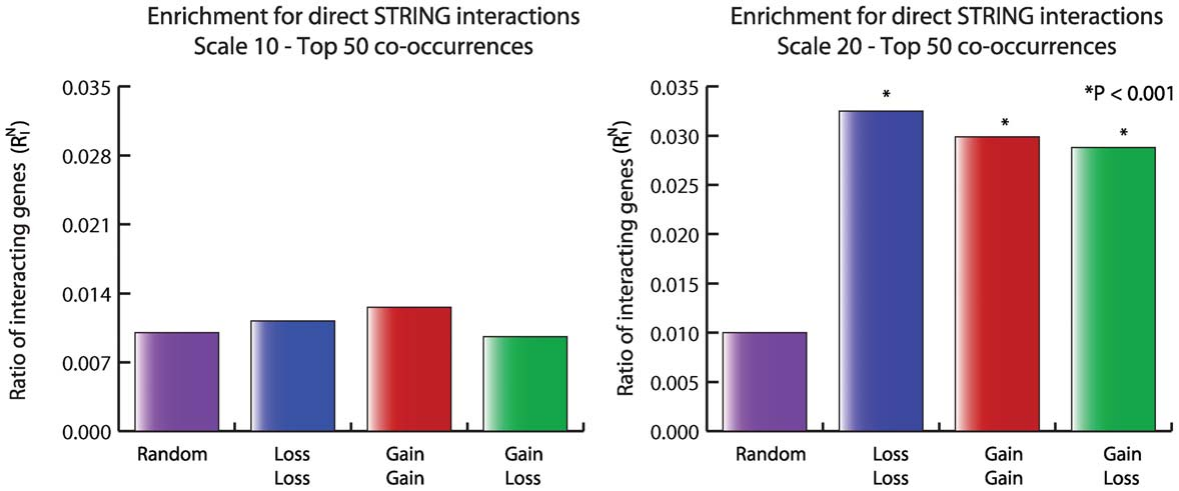
Significance of finding direct interactions in co-occurring genomic loci. For two scales the top 50 co-occurring gene lists for the gain-gain, loss-loss and loss-gain situations were compared to a random set of 100 pairs of genomic loci. For each genomic pair two gene sets were queried for direct interactions using the STRING database. Significance was ascertained using Fisher's exact test on the ratios between all genes and the interacting genes for the co-occurrence gene sets versus the random gene set.

While finding enrichment for cancer genes is an encouraging result, this does not explain the possible cooperation between two loci. We expect that the co-occurring loss of two regions points to a functional relationship between the constituents of the genomic loci. A co-occurrence between two genomic regions can point to many different kinds of interactions between the genes residing in both regions, e.g. biochemical interactions of the protein products or functional collaboration of two cancer genes in tumorigenesis. We therefore decided to employ interaction data to shed further light on the genes present in the co-occurring regions. We translated the co-occurring pairs of genomic loci to pairs of gene sets, and we investigated the functional relationships of their protein products using the STRING database [10] (version 8.1). The STRING model weighs functional associations between genes based on several different sources of evidence, among which: biochemical interaction, joint presence in a pathway, high-throughput interaction experiments, text mining and interactions of homologs in other species. To find a functional relationship between two co-occurring regions we looked for a direct interaction in the STRING database between the two gene-sets defined by our co-occurrence analysis. To determine whether the number of observed interactions is significant, we compared the number of direct interactions found between genes located in the top 50 co-occurring regions to a set of randomly chosen pairs of genomic loci. The metric we used to determine significance is the ratio between the number of interacting genes and the total number of genes found on the genomic loci. A p-value for enrichment for direct interactions was calculated using a two-tailed Fisher's exact test. Results are shown in Figure 4. As can be seen, the only analysis that resulted in an enrichment of functional interactions is Scale 20, for all three situations. We found no enrichment for interacting protein coding genes on Scale 2 (not shown) and Scale 10. Since we evaluated gene sets in a window one-third the size of the analysis-scale we may be under-estimating the size of the co-occurring loci and the larger Scale 20 actually captures the size of the aberrations best.

In order to keep control of the complexity, we considered in our co-occurrence analysis only radially symmetric kernels, i.e. Gaussian kernels with diagonal, equal variance covariance matrices. This implies that asymmetric co-occurring regions – where a small locus co-occurs with a large locus – will not be optimally detected. Since an asymmetric co-occurring region typically consists of a series of symmetric co-occurring regions detected on a smaller scale (just like a rectangle can be constructed from a collection of squares), we set out to construct larger co-occurring regions from the results of the smaller scales using a hierarchical clustering approach. For details see Supplemental Figure S1.

**Figure 5:**
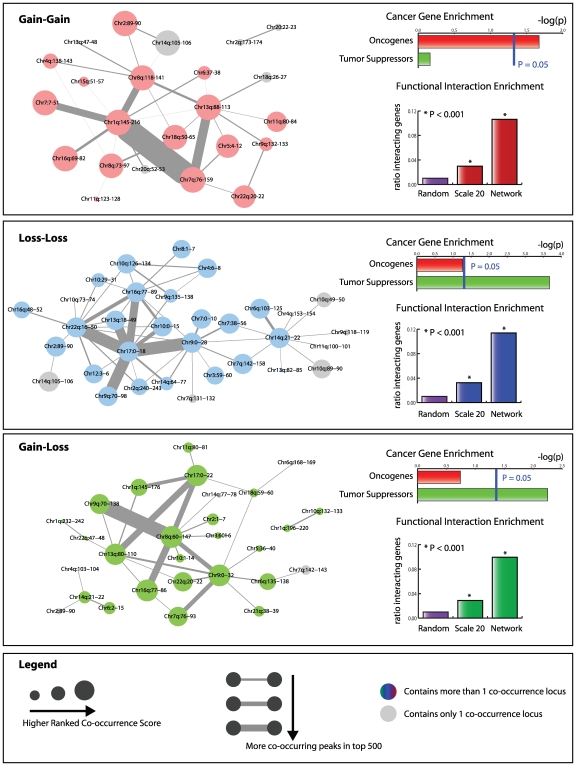
Networks of co-occurring gain and loss. The networks that result from hierarchical clustering of Scale 2 results are shown in different panels. Each panel represents either the gain-gain, loss-loss or gain-loss analysis. The resultant network is visualized using the Cytoscape software package (www.cytoscape.org). Edge thickness scales according to the number of co-occurrence links found between the two genomic loci. The size of the nodes is proportional to the highest rank found among the different individual loci that constitute a node. If only one genomic location is present in a node, i.e. this location did not cluster with any other locations, it is colored gray. The cancer gene enrichment among all genes mapping to the locations described by the nodes is shown in the top right hand corner. P-values are determined by Fisher's Exact test. The functional interaction enrichment of all genes between nodes that are linked with an edge is represented in the lower right hand corner of each panel. P-values are determined using Fishers' Exact test, with randomly generated pairs of loci representing the null hypothesis.

Briefly, we collected the loci involved in the top 500 co-occurrences of the Scale 2 analysis. This resulted in 1000 genomic loci. For each pair of loci, we calculated the genomic distance in base pairs. The distance between two loci on different chromosome arms was set to a default high value (1 * 10^8^). This resulted in a 1000×1000 distance matrix. On this distance matrix we performed single linkage hierarchical clustering. The resulting dendrogram was cut at 1 * 10^7^ bp (5 kernel widths). The resulting clusters are unique genomic loci and were represented as nodes in a graph. Clusters were then linked if a co-occurrence was found between individual loci of different clusters. These links are represented as edges in a graph. The result of the clustering analysis is shown in Figure 5.

### A network view of co-occurring copy number changes

As can be seen in Figure 5 we were able to construct a network of co-occurring copy number changes for the gain-gain, loss-loss and gain-loss situations. As expected, the gain-gain and loss-loss networks show enrichment for oncogenes and tumor suppressor genes, respectively. The gain-loss network only shows enrichment for tumor suppressors. The percentage of genes involved in functional interactions between the nodes that are linked in the graph vastly exceeds the functional interaction enrichment found in the single scale 20 Mb analyses. At least 11% of the genes present in the genomic locations - represented by the nodes in the graphs - have high confidence (>0.9) annotated functional interactions along the edges as revealed by STRING analysis.

**Figure 6:**
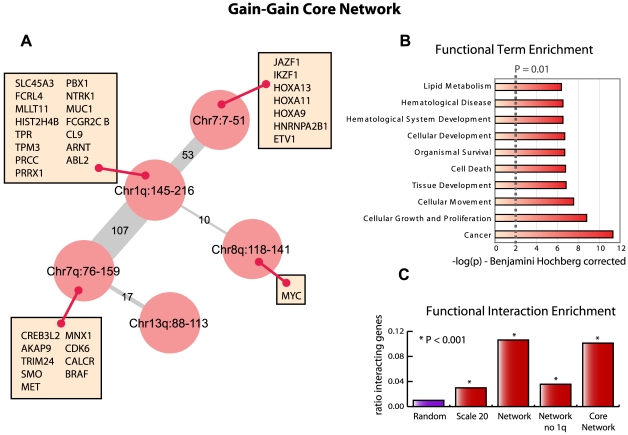
The gain-gain core network. a. The reduced core network for the gain-gain analysis obtained by pruning all edges with less than 5% support in the top 500 list of the Scale 2 analysis. Edge thickness and label represent the number of functional interactions between genes associated with the nodes being connected based on the STRING database. The oncogenes as defined by the Cancer Gene Census that map within the regions defined by the nodes are shown in rectangular insets. b. Representation of the 10 most enriched Ingenuity terms associated with the entire collection of genes in the core network that have a STRING interaction along the edges. The x-axis shows the −log transformed p value, corrected by the Benjamini Hochberg procedure as implemented in the Ingenuity software. c. Functional interaction enrichment is shown as a bar graph, which represent the ratio of interacting genes with respect to the total number of genes. P-values are determined using a Fishers' Exact test with randomly selected pairs of loci representing the null hypothesis.

**Figure 7:**
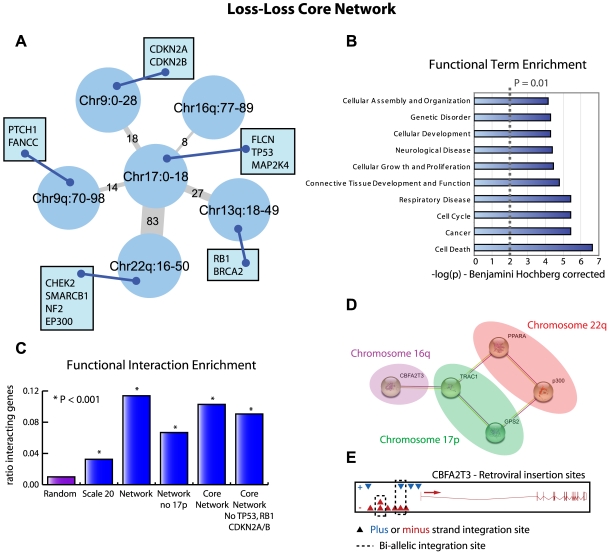
The loss-loss core network. a. The reduced core network for the loss-loss analysis determined by pruning all edges with less than 5% support in the top 500 list of the Scale 2 analysis. Edge thickness and label represent the number of functional interactions between genes associated with the nodes being connected based on the STRING database. The tumor suppressor genes as defined by the Cancer Gene Census that map within the regions defined by the nodes are shown in rectangular insets. b. Representation of the 10 most enriched Ingenuity terms associated with the entire collection of genes that have a STRING interaction between the 17p region and 9p, 9q, 13q, 16q or 22q as determined by the Ingenuity software. The x-axis shows the −log transformed p value, corrected by the Benjamini Hochberg procedure as implemented in the Ingenuity software. c. Functional interaction enrichment is shown as a bar graph, which represent the ratio of interacting genes with respect to the total number of genes. P-values are determined using a Fishers' Exact test with randomly selected pairs of loci representing the null hypothesis. d. A functional interaction network around the nuclear co-repressor *NCOR1* (also known as *TRAC1*) is shown. This network is a part of the network of interactors derived from the 17p interacting regions after removal of the canonical cancer genes *TP53*, *RB1*, *CDKN2A* and *CDKN2B* from the analysis. e. Illustration of the retroviral insertions mapped near *CBFA2T3*, recovered in a large screen of MuLV retroviral mutagenesis [11]. Insertions are shown as triangles. Blue triangles indicate insertions in the direction of transcription (plus), red triangles indicate insertions in the anti-transcription direction (minus). Insertions linked by dashed boxes are bi-allelic integrations recovered from the same tumor.

The thickness of the edges in the graphs shown in Figure 5 indicates how often a co-occurrence was found in the top 500 of the Scale 2 analysis. Several edges were strongly supported by co-occurrences in the top 500. These strongly supported edges were always associated with loci that were ranked high in the co-occurrence list (as indicated by node size). The nodes that are associated with these highly represented edges seem to form an important subgraph. To reveal these subgraphs, we removed all edges supported by less than 5% of the top 500 co-occurrences. For brevity and simplicity we only consider the gain-gain and loss-loss networks. This resulted in the two core networks shown in Figures 6 and 7.

### The gain-gain core network

The edge thickness of the gain-gain core network shown in Figure 6 represents the number of functional interactions found using the STRING database between genes that map within the loci described by the nodes. To determine the common denominator among the interacting genes, we employed Ingenuity Pathway Analysis (IPA; Ingenuity Systems) to perform a functional enrichment analysis on all genes residing within the gain-gain core network. This revealed strong enrichment for processes involved in cancer (Figure 6b). From Figure 6a it is immediately apparent that most of the functional interactions are found between 1q and 7p/q. If we remove the 1q node from the entire network described in Figure 5 the enrichment for functional interaction drops dramatically (Figure 6c). Therefore, we hypothesize that the co-occurring gain between 1q and 7p/q is the most important effect in the gain-gain analysis in this dataset. This is strengthened by the fact that almost all known oncogenes within the entire network map to 1q, 7p or 7q (Figure 6a). The well-studied canonical oncogene *M*Y*C* maps to 8q and is not a determining hub in the gene interaction network as constructed by STRING.

### The loss-loss core network

The loss-loss core network is shown schematically in Figure 7a. A loss of approximately 18 megabases on chromosome 17p appears to be a central hub, which is co-lost with several other genomic loci. These loci show a very high enrichment of genes that interact with 17p, and of the six loci, four contain multiple known tumor suppressor genes. A functional enrichment analysis of all genes residing on loci co-lost with 17p, reveals many cancer-related processes (Figure 7b), suggesting that the interacting genes are most likely also the cancer-relevant genes. If we remove 17p from the original network we see a large decrease in the percentage of genes involved in functional interactions (Figure 7c) confirming the importance of 17p in the loss-loss network.

### The co-occurring losses involving 17p might target non-canonical cancer genes

One of the most intensively studied cancer genes, *TP53*, resides in the 17p locus. Furthermore, the canonical cancer gene *RB1* and the *CDKN2A/B* locus are present in two of its co-lost regions. Since these are well known tumor suppressors, and therefore the subject of thousands of research papers, they might constitute the bulk of the functional relationships in our analysis. To test this hypothesis, we excluded these four genes and repeated the interaction analysis of the core network. As can be seen in Figure 7c, the enrichment is only slightly lower without the canonical genes, suggesting that the functional relationship between the co-occurring losses on 17p and the other loci are driven by other genes.

We investigated the remaining 113 interactors for any interesting interactions that might be a target of this collection of co-occurring losses. Within the total network of interactors we found a sub-network centered on the nuclear co-repressor *NCOR1* (*TRAC1*) (Figure 7d). This interaction network included – besides *NCOR1* – the peroxisome proliferator-activated receptor alpha (*PPARA*), the MAPK pathway suppressor GPS2, the nuclear co-activator (and known tumor-suppressor) *p300* and a gene of unknown function, *CBFA2T3*. All interactions found are based on physical binding and co-occurrence in Pubmed abstracts.

### Combining co-occurrence data with insertional mutagenesis data

To see whether we could find more information regarding the putative tumor suppressor function of the different interactors, we tested if we could corroborate our findings with data from a large retroviral insertional mutagenesis (IM) screen where hematopoietic tumors were induced through Murine Leukemia virus (MuLV) infection of wild-type mice or *Trp53* or *p19-ARF* deficient mice [11]. An illustration of the retroviral insertions sites near *Cbfa2t3* is shown in Figure 7d. Although *Cbfa2t3* was not flagged as a common integration site, several viral integrations near this gene were found. Remarkably, two individual tumors harbored a bi-allelic integration near the transcription start site of *Cbfa2t3*, suggesting functional inactivation of this candidate tumor suppressor gene. Indeed, bi-allelic integration is thought to be a hallmark of tumor suppressor genes in IM screens [12].

Given that we find this sub-network of interactors in a co-occurring network of DNA copy number losses and the recovery of inactivating insertions in a retroviral IM screen, we conclude that this network might be a putative tumor suppressor network.

## Discussion

In this study we present a genome-wide analysis for finding collaborating DNA copy number changes on different chromosomes. Using our 2D kernel convolution framework we can score and find co-occurring DNA copy number changes in a high quality, high resolution aCGH dataset. Using a dataset of hematological cell lines we are able to recover DNA copy number alterations specific to the cell lineage of the samples. Furthermore we uncover cancer-related networks of co-occurring DNA copy number changes.

### Previous work on co-occurring copy number changes

Several studies have investigated concerted copy number changes in aCGH data. In studies on lung cancer [13] and ovarian cancer [14] the authors performed a post-hoc co-occurrence analysis on genomic locations that were found to be significantly altered in a one-dimensional analysis. A more integrated effort to analyze relations between CNAs in brain cancer was published recently [15]. Although this study scores systematically for co-aberration, it is limited in resolution as it employs cytobands as the genomic unit within which aberrations are scored. Cytobands are relatively arbitrarily determined entities and are quite heterogeneous in size. Furthermore this approach is dependant on converting the continuous-valued copy number data to discrete copy number calls. This results in loss of important information since it removes the possibility of weighting the intensity of a CNA. In contrast, our approach is able to correct for unequal probe distances, enabling us to perform our analysis on a very high (20 kbp) resolution. In addition, our scoring method not only incorporates the sign of the copy number change, but also its intensity and the concomitant CNAs within the immediate genomic neighborhood.

### Determining significance of co-occurrences

The output of our analysis does not include a measure of significance. Constructing a background distribution based on permutations of the DNA copy number data would mean re-running our analysis thousands of times, a task which remains computationally infeasible at this stage. Furthermore, the multiple-testing problem would have to be properly addressed, given that the number of tests is the square of the number of grid points in the 2D space. Due to the complexity of the analysis procedure (minimum operation and kernel smoothing) the definition of an analytical null distribution has remained elusive. Therefore, we have chosen to work with top *n* results, residing in the extremes of the results distribution, thus minimizing the chance of including false positives. The top *n* lists allowed us to generate workable results which we have validated extensively with other sources of evidence.

### Analytical challenges

While we were able to use a distributed computing solution for our analysis, we were fortunate to have the required computational architecture at our disposal. Since the problem basically consists of repeating the same action many times it could be well-suited to software optimization or a hardware based solution where the most time-consuming actions are handled by a dedicated processing unit.

When looking for areas in the 2D pair-wise space highly enriched for co-occurrence scores we convolve this space with a 2D-Gaussian kernel. The sigma parameter of this function is a representation of the size of the aberrations we expect to recover. Currently we make the implicit assumption that the co-occurring aberrations have the same size by using a symmetric kernel for the convolution. This could be relieved by allowing for an asymmetrical (ellipsoid) Gaussian kernel for all combinations of scales used. Clearly, this comes at the cost of increased computational complexity. Here we resolve this issue by concatenation of the results obtained in a small scale. In this way we can recover co-occurring losses of different sizes that give a better enrichment for functional interactions when combined with the single peaks obtained in a higher scale analysis.

### Gene dosage effects on a large number of genes

In our analysis of a set of cell lines derived from hematological malignancies we found enrichment of cancer related genes and functional interactions in co-occurring DNA copy number changes. Our results suggest that tumorigenesis requires elimination of multiple gatekeeper genes and gain of multiple oncogenes as demonstrated by the presence of many functional interactions between the loci in the gain-gain and loss-loss core networks.

Haploinsufficiency is a well known characteristic of several tumor suppressor genes, where simple reduction of gene dosage by loss of gene copies at the DNA level can already promote oncogenic transformation [16]. It is conceivable that changes in gene dosage of multiple interconnected genes involved in cancer-related processes such as cell cycle, DNA repair and signaling can also weaken a cells defense against uncontrolled cell proliferation. In this case, heterozygous loss or gain of large genomic regions, such as the ones identified in this study, might effectively sensitize cells to become tumorigenic.

### 17p as a central player in co-occurring losses

We show that the 17p loss and its co-lost regions are highly enriched for functional relationships, which are not fully explained by the presence of the *TP53* gene, often thought to be the single target of this deletion [17]–[19]. Although *TP53* is no doubt an important target of the DNA copy number loss, our analysis indicates that the concomitant loss of other genes near *TP53*, as well as co-occurring losses on the other genomic loci may together account for the full tumorigenic effect.

Loss of the loci on 17p, 9p, 9q, 13q, 16q and 22q has been reported previously for several types of hematological malignancies represented in our dataset [20]–[23]. The picture that emerges from this analysis of collaborative aberrations is that many of the reported losses collaborate with the frequently occurring 17p loss as a central hub. We don't recover co-occurring losses among the spoke loci in the core network. This could suggest that the non-17p regions form subsets of co-occurring losses with 17p, whose interconnections themselves do not occur frequently enough in the top 500 co-occurring losses we investigated.

### *NCOR1* and its interactors

Not all of the gene-gene interactions defined by the 17p network involve the well-known canonical cancer genes *TP53*, *RB1* and *CDKN2A* (*INK4a/ARF*). We found one sub-network of genes around *NCOR1* which might be an example of other tumor suppressor genes that are affected by the concerted loss of these genomic loci.

The hub of this interaction network, *NCOR1*, is a well-known transcriptional co-repressor that associates in a ligand-independent manner with nuclear receptors [24]. It is responsible, together with the closely related factor *SMRT*, for recruitment of HDAC proteins to the DNA to induce transcriptional silencing. Its role in cancer is not well-established. *NCOR1* null mice die in early embryogenesis [25]. A dominant-negative mutant of *NCOR1* is known to increase proliferation in hepatocytes [26] and more recently it has been shown that *NCOR1* decreases *AKT* phosphorylation, thus countering its pro-survival signal [27]. It would seem that specific loss - or at least decrease in gene dosage of *NCOR1* - might increase proliferation and promote survival.

All interactions between *NCOR1* and its partner genes (*PPARA*, GPS2 *a*nd *CBFA2T3*) have been based on co-occurrence in PubMed abstract and true physical binding [28]–[31]. *CBFA2T3* is a close relative of *ETO*, which is a target of the recurrent *AML1*-*ETO* translocation that occurs in acute myeloid leukemia. It has been shown that the fusion gene *AML1-ETO* actually interferes with the *CBFA2T3-NCOR1* interaction, and that its oncogenic effect derives from that inhibition [31]. In a retroviral insertional mutagenesis screen in mice, *Cbfa2t3* is recurrently targeted by bi-allelic retroviral integrations, which are predicted to cause functional inactivation of *Cbfa2t3* [11]. *PPARA* is a member of the Peroxisome proliferator-activated receptors, and has been implicated in hepatocellular carcinoma development in rodents [32]. Since other members of this family, such as *PPARG*, exhibit a tumor suppressor-like phenotype, it is possible that *PPARA* can act as a tumor suppressor in hematological malignancies. *GPS2* is a known suppressor of *JNK* signaling [33], which is one of the constituents of the *MAP* kinase signaling pathway. Deregulation of this pathway is a well-known phenomenon in cancer [34]. Taken together with the association between *NCOR1* and the known tumor suppressor *p300*, our data suggest a selective advantage for loss of multiple constituents that interact with *NCOR1* since they all may have tumor suppressor-like activities.

Many studies focus on a single hematological malignancy in which a single combination of aberrations might be important [19],[35],[36]. Since we examine a large panel of samples derived from many different hematological malignancies, our results might not specifically apply to any single type of lymphoma or leukemia. They might hint at more general processes that are important for the tumors to arise and maintain themselves. However, one should not forget that this analysis is based on a panel of cell lines, which may have adapted to in vitro tissue culture conditions by acquiring additional aberrations that are rarely found in real tumors in patients. Furthermore, given the fact that we examine copy number changes it might be worthwhile to analyze a highly genomically unstable tumor type, such as *BRCA1/2*-related breast cancer.

### Conclusions

We have developed a method for genome-wide analysis of collaborating DNA copy number changes and their corresponding networks. Using this approach we have identified a loss-loss network centered around a region on human chromosome 17p. This network is highly enriched for functional relationships and hints at a more complex system of tumor suppression in which many different genes are affected simultaneously to induce cancer. We show one example of a sub-network around the nuclear co-repressor *NCOR1* that may be a novel network of tumor suppressor genes that are affected by the observed co-occurring losses. The observation that DNA copy number changes may affect gene dosage of larger numbers of cancer-relevant genes deviates from the classical view where mutations in a few (5–7) cancer genes lead to tumor development. Our data support the notion of cancer-related networks or pathways, where multiple collaborating genes are deregulated simultaneously to induce oncogenesis. Such a network view of oncogenesis is an important step towards developing effective drug targets because it increases the number of potential targets. However, this view also implies that multiple molecules need to be targeted simultaneously in order to achieve optimal therapy response and to reduce the risk of therapy resistance.

## Materials and Methods

### Transformation of probe-measurements to genomic grid

Datasets consisting of array-based copy number measurements are continuously increasing in size. If probe level interactions are evaluated, the analysis space is of dimensionality 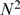 for 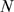 probes on the genome. As a result, the analysis time and memory usage will also increase quadratically with the number of probes. Instead of a grid positioned at the genomic positions of the probes, we employ an equally spaced genomic grid as a basis for all subsequent steps. The distance between grid-points is a user-defined variable, and will determine the finest resolution of the outcome and computational efficiency. We have performed all analyses using a genomic grid with a grid spacing of 20 Kb. Given a genome of 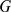 base pairs and a grid spacing of 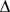, this results in 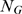 grid positions, with 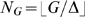, where 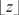 represents the integer part of the real number, 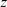. The grid positions can be represented in the following row vector: 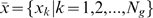, where 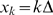.

Let the aCGH profile of the 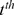 tumor be represented by the following row vector of probe measurements: 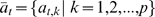, with 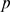 being the number probes. Let the midpositions of the probes be located at 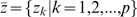. To employ the genomic grid we need to compute, for each aCGH array, the value of the aCGH profile on the grid points. We achieve this by performing, for the 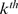 grid position, 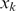, a kernel-weighted regression of all probe values situated in the range 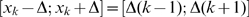, employing a triangular kernel centered at 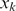, with maximal amplitude of 1 and width of 2 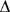. More specifically, the interpolated copy number aberration at the 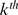 grid position is given by:
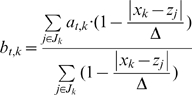
(1)Here the set 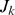 is the set of probe positions such that 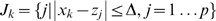. The interpolated copy number profile of the 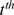 tumor is represented by the row vector: 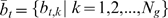. The complete dataset of 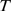 tumors is refopresented by the matrix 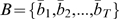, where the probe values of the 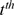 tumor constitute the 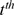 row of matrix 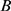.

### Separating gains and losses

Negative and positive log2 values respectively denote loss or gain of DNA in the test sample versus the reference sample. We regard both situations separately, which prevents the negative and positive values cancelling each other through summation later in the algorithm. We separate gains and losses by only retaining grid positions with positive values for the gains or negative values for the losses. The absolute values of the separated matrices are then used in the downstream steps. The remaining grid positions are set to zero. More specifically, the gains matrix, 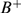 is given by 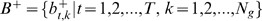, with
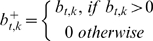
(2)Similarly, the loss matrix, 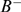 is given by 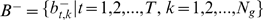 with
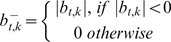
(3)Because we treat gains and losses separately we have four different co-occurrence situations to be considered given two loci on the genomic grid: i) gain/gain, ii) gain/loss, iii) loss/loss and iv) loss/gain. So, when evaluating the co-occurrence of loci 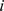 and 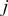, we will evaluate the behavior of i) columns 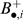 and 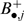 for gain/gain; ii) columns 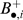 and 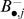 for gain/loss; iii) columns 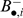 and 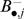 for loss/loss and iv) columns 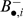 and 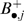 for loss/gain. (Here 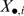 is the 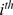 column of matrix 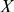). All subsequently described steps will be performed for these four situations separately, where 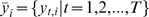 and 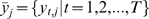 will be employed as shorthand for the abovementioned column vectors of interpolated copy number values associated with genomic grid positions 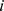 and 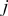, respectively.

### Co-occurrence score

The first component of the co-occurrence score is the continuous variant of the AND Boolean logic function: the minimum operation. For two grid points, 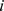 and 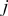, the sum across all tumors of the minimal probe value per tumor at 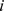 and 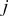, 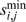, is calculated as follows:
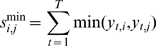
(4)These values are aggregated in a matrix, 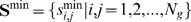. If we only use the minimum as a scoring function, those grid positions that are ubiquitously aberrated will always receive a high score, regardless of the aberration pattern in the other grid position. Two regions that are aberrated ubiquitously in all tumors are undoubtly important to the tumor but they are not necessarily functionally related. They might be a hallmark of the particular disease under study, but show no direct functional interaction. To prevent these ubiquitously aberrated regions from dominating the analysis and to detect those regions that represent functional co-occurrences, we weigh the minimum score computed above with the covariance of the interpolated probe values at the two grid positions 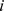 and 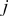,
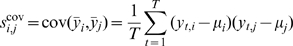
(5)where 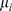 and 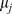 are the expected values of the probe values at grid position 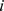 and 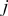 across tumors, respectively (i.e. 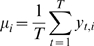 and 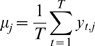). These values are aggregated in a matrix, 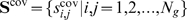. We combine both the minimum matrix and the co-variance matrix by element-wise multiplication to form the co-occurrence score matrix, 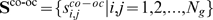, with
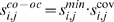
(6)


### Kernel convolution

Since we believe the co-occurrence score to be a smooth variable, and since neighboring co-occurrence values can therefore be employed to reduce the noise locally, we convolve the co-occurrence score matrix with an isotropic 2D Gaussian kernel function. In practice this implies sampling the 2D Gaussian kernel function on a square grid consisting of 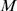
×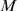 genomic grid positions and then performing the convolution of this sampled kernel function with the co-occurrence score matrix. The sampled isotropic 2D Gaussian function is defined as 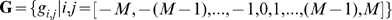, with
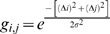
(7)The standard deviation of the isotropic Gaussian, 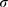, determines the scale of the analysis. Since the Gaussian quickly decays we set 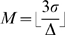, allowing contributions from 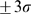, convolving with a finite kernel with minimal loss in accuracy. The scale of an analysis is therefore defined as 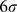. The scales employed in this study are: 2 Mb (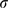 = 1/3 Mb), 10Mb (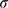 = 5/3 Mb) and 20Mb (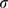 = 10/3 Mb). Before the convolution step, we pad the co-occurrence matrix by mirroring the true data at each chromosome boundary and each centromere. By convolving the appropriately padded co-occurrence score matrix and the sampled 2D Gaussian function the Convolved Co-occurrence Matrix (CCM) is obtained:
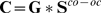
(8)with 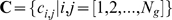, 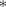 as the convolution operator and
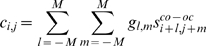
(9)This matrix is a representation of the amount of co-occurrence between two locations on the genome. We calculate a CCM-matrix for each possible combination of chromosome-arms and for each of the four combinations of gains and losses listed above.

### Distributed computing

With 39 unique chromosome arms in the human genome (disregarding the p-arms of the acrocentric chromosomes and the sex chromosomes), three different scales and 4 triangular pair-wise matrices to evaluate (loss-loss, gain-gain, gain-loss and loss-gain) we compute 8892 different CCMs. To solve this problem computationally we used a large distributed computing cluster. Our choice of resolution of the genomic grid was bounded by the memory present on the nodes. We set 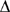 to 20000 base pairs, which is the lowest value still allowing the largest chromosome-arm pair to be successfully computed on one computing node.

### Downstream analyses

For each CCM we determine the top N peaks for each combination of gains and losses. The n^th^ peak represents two co-occurring loci, 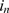 and 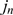, and the location of the peak is defined by two co-ordinates on the genomic grid: 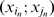. For each locus, we define a region of interest of size 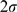 centered on 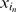 and 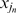, respectively. We define this small region of interest to only select regions that are very near to the actual peak. To investigate the co-occurrence for functional relationships, we extract, for each of the co-occurring loci, the genes present in the regions of interest. More specifically, we define, for loci 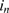 and 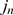, the associated gene sets 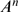 and 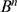, where
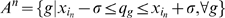
(10)and
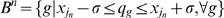
(11)Where 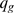 is the position of gene 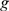, which we chose to be the mid-position of the gene. The genesets were established by a BioMart query from the Ensembl database. We restricted ourselves to the bio type ‘protein_coding’.

### Cancer Gene Census (CGC) enrichment

The list of CGC genes was obtained from the CGC website (http://www.sanger.ac.uk/genetics/CGP/Census/). The reference list of all genes was retrieved from the Ensembl website, with a filter to keep only genes with bio-type = ‘protein_coding’. This left 18840 genes. All CGC genes that could not be mapped back to the reference gene set were excluded. The CGC genes that were annotated as ‘recessive’ were used as the tumor-suppressor genes and ‘dominant’ as oncogenes. Enrichment for all CGC genes, the tumor-suppressor subgroup and the oncogene subgroup in the gene sets determined by the co-occurrence analysis was calculated using a Fisher's exact test.

### Analysis of functional relationships

The set of pairs of interacting genes which are such that one gene is associated with locus 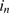 and the other gene of the pair with locus 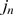 is then defined as
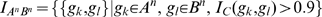
(12)Where 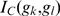 represents the confidence of interaction, according to the STRING database, between genes *g_k_* and *g_l_*. We then determine all gene lists of interactors for the top N peaks of a given co-occurrence analysis, i.e.:
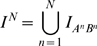
(13)For each of the top *N* co-occurring loci, we also determine the total number of genes in the regions of interest of those loci. So, for loci 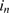 and 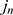 we define the set:
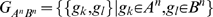
(14)The total number of genes associated with the top *N* co-occurring loci is then given by
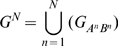
(15)The interaction ratio, 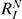, is then defined as
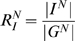
(16)where 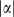 denotes the cardinality of set 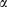. As a control we randomly pick size-matched locations for all co-occurring regions 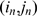 in the top N and repeat the process for recovering interactions. For 100 randomly chosen co-occurring regions we calculate the resulting 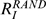. A Fisher's exact test is then used to asses the significance of enrichment of 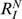 versus 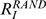.

### Hierarchical clustering of co-occurrence loci

For all pairs of co-occurring loci, 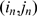, present in the top *N* of an analysis,
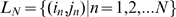
let the set of loci representing the first and second member of the co-occurrence locus be defined as
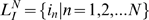
and
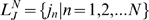
respectively. Given that the pairs of genomic locations corresponding to the top *N* co-occurring loci are given by
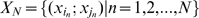
we define the set of genomic locations loci involved in co-occurrences as
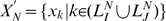
For each possible pair of locations 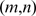 in 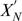 the genomic distance is aggregated in matrix 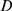:
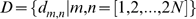
(18)Where 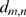 is defined as:
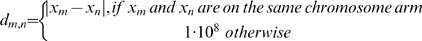
(19)We perform hierarchical clustering on matrix 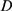 using single linkage hierarchical clustering. Leaf nodes are assigned to clusters using a distance cutoff of 10^7^ bp (10Mb). Clusters are represented as nodes in a graph. Edges between nodes are drawn if any co-occurrence relationship is found between loci present in the nodes.

### Data description

The case we subjected to analysis was a dataset containing 105 cell-lines derived from hematological origin. The aCGH measurements were done on 1.8 million probe Affymetrix SNP 6.0 arrays. After data pre-processing we were left with 95 samples. These cell lines are a subset of the Cancer Genome Project cancer cell line project (http://www.sanger.ac.uk/genetics/CGP/CellLines/). A list of the cell lines included in this dataset can be found in Dataset S1.

## Supporting Information

Figure S1 Hierarchical clustering of small scale CCM peaks. a. All peaks present in the top 500 that report a co-occurrence between these chromosome arms can be represented as a table of 1000 genomic loci, with 500 pairwise co-occurrence relationships. b. The 1000 genomic loci are individually clustered based on their genomic distance. The distance between two loci is the absolute difference in basepairs if both are mapped to the same chromosome arm and a high constant value if not. Single linkage is used to grow a dendrogram from these distances. Clusters of genomic loci are defined by aggregating all children under a certain set cutoff distance, 10Mb in this case. c. The clusters defined in b. are represented as nodes in a network. Edges are drawn between nodes if a co-occurrence relationship exists between any genomic loci assigned to the clusters.
(1.14 MB EPS)

Dataset S1 This file contains information about the cell lines used in this study.
(0.03 MB XLS)

## References

[1] Hanahan D, Weinberg RA (2000). The Hallmarks of Cancer.. Cell.

[2] Michor F, Iwasa Y, Nowak M (2004). Dynamics of cancer progression.. Nat Rev Cancer.

[3] de Ridder J, Kool J, Uren A, Bot J, Wessels L (2007). Co-occurrence analysis of insertional mutagenesis data reveals cooperating oncogenes.. Bioinformatics.

[4] Thomas R, Baker A, DeBiasi R, Winckler W, LaFramboise T (2007). High-throughput oncogene mutation profiling in human cancer.. Nat Genet.

[5] Rajagopalan H, Nowak M, Vogelstein B, Lengauer C (2003). The significance of unstable chromosomes in colorectal cancer.. Nat Rev Cancer.

[6] Sieber O, Heinimann K, Tomlinson I (2003). Genomic instability - the engine of tumorigenesis?. Nat Rev Cancer.

[7] Pinkel D, Albertson DG (2005). Array comparative genomic hybridization and its applications in cancer.. Nat Genet.

[8] Schmitz R, Renné C, Rosenquist R, Tinguely M, Distler V (2005). Insights into the multistep transformation process of lymphomas: IgH-associated translocations and tumor suppressor gene mutations in clonally related composite Hodgkin's and non-Hodgkin's lymphomas.. Leukemia.

[9] Futreal PA, Coin L, Marshall M, Down T, Hubbard T (2004). A census of human cancer genes.. Nat Rev Cancer.

[10] Jensen L, Kuhn M, Stark M, Chaffron S, Creevey C (2009). STRING 8–a global view on proteins and their functional interactions in 630 organisms.. Nucl Acids Res.

[11] Uren AG, Kool J, Matentzoglu K, de Ridder J, Mattison J (2008). Large-Scale Mutagenesis in p19ARF-and p53-Deficient Mice Identifies Cancer Genes and Their Collaborative Networks.. Cell.

[12] Suzuki T, Minehata K, Akagi K, Jenkins NA, Copeland NG (2006). Tumor suppressor gene identification using retroviral insertional mutagenesis in Blm-deficient mice.. The EMBO Journal.

[13] Chitale D, Gong Y, Taylor BS, Broderick S, Brennan C (2009). An integrated genomic analysis of lung cancer reveals loss of DUSP4 in EGFR-mutant tumors.. Oncogene.

[14] Haverty P, Hon L, Kaminker J, Chant J, Zhang Z (2009). High-resolution analysis of copy number alterations and associated expression changes in ovarian tumors.. BMC Medical Genomics.

[15] Bredel M, Scholtens D, Harsh G, Bredel C, Chandler J (2009). A Network Model of a Cooperative Genetic Landscape in Brain Tumors.. JAMA.

[16] Quon K, Berns A (2001). Haplo-insufficiency? Let me count the ways.. Genes Dev.

[17] Dohner H, Fischer K, Bentz M, Hansen K, Benner A (1995). p53 gene deletion predicts for poor survival and non-response to therapy with purine analogs in chronic B-cell leukemias.. Blood.

[18] Drach J, Ackermann J, Fritz E, Kromer E, Schuster R (1998). Presence of a p53 Gene Deletion in Patients With Multiple Myeloma Predicts for Short Survival After Conventional-Dose Chemotherapy.. Blood.

[19] Seifert H, Mohr B, Thiede C, Oelschlagel U, Schakel U (2009). The prognostic impact of 17p (p53) deletion in 2272 adults with acute myeloid leukemia.. Leukemia.

[20] Stilgenbauer S, Bullinger L, Lichter P, Dohner H (2002). Genetics of chronic lymphocytic leukemia: genomic aberrations and VH gene mutation status in pathogenesis and clinical course.. Leukemia.

[21] Heerema NA, Sather HN, Sensel MG, Liu-Mares W, Lange BJ (1999). Association of Chromosome Arm 9p Abnormalities With Adverse Risk in Childhood Acute Lymphoblastic Leukemia: A Report From the Children's Cancer Group.. Blood.

[22] Berglund M, Enblad G, Flordal E, Lui WO, Backlin C (2002). Chromosomal Imbalances in Diffuse Large B-Cell Lymphoma Detected by Comparative Genomic Hybridization.. Modern Pathology.

[23] Strefford JC, Worley H, Barber K, Wright S, Stewart ARM (2007). Genome complexity in acute lymphoblastic leukemia is revealed by array-based comparative genomic hybridization.. Oncogene.

[24] Horlein A, Naar A, Heinzel T, Torchia J, Gloss B (1995). Ligand-independent repression by the thyroid hormone receptor mediated by a nuclear receptor co-repressor.. Nature.

[25] Jepsen K, Hermanson O, Onami T, Gleiberman A, Lunyak V (2000). Combinatorial Roles of the Nuclear Receptor Corepressor in Transcription and Development.. Cell.

[26] Feng X, Jiang Y, Meltzer P, Yen P (2001). Transgenic Targeting of a Dominant Negative Corepressor to Liver Blocks Basal Repression by Thyroid Hormone Receptor and Increases Cell Proliferation.. J Biol Chem.

[27] Furuya F, Guigon C, Zhao L, Lu C, Hanover J (2007). Nuclear Receptor Corepressor Is a Novel Regulator of Phosphatidylinositol 3-Kinase Signaling.. Mol Cell Biol.

[28] Yoon HG, Chan DW, Huang ZQ, Li J, Fondell JD (2003). Purification and functional characterization of the human N-CoR complex: the roles of HDAC3, TBL1 and TBLR1.. EMBO J.

[29] Yan Z, Jetten A (2000). Characterization of the Repressor Function of the Nuclear Orphan Receptor Retinoid Receptor-related Testis-associated Receptor/Germ Cell Nuclear Factor.. J Biol Chem.

[30] Dowell P, Ishmael J, Avram D, Peterson V, Nevrivy D (1999). Identification of Nuclear Receptor Corepressor as a Peroxisome Proliferator-activated Receptor alpha Interacting Protein.. J Biol Chem.

[31] Ibanez V, Sharma A, Buonamici S, Verma A, Kalakonda S (2004). AML1-ETO Decreases ETO-2 (MTG16) Interactions with Nuclear Receptor Corepressor, an Effect That Impairs Granulocyte Differentiation.. Cancer Res.

[32] Michalik L, Desvergne B, Wahli W (2004). Peroxisome-proliferator-activated receptors and cancers: complex stories.. Nat Rev Cancer.

[33] Zhang J, Kalkum M, Chait B, Roeder R (2002). The N-CoR-HDAC3 Nuclear Receptor Corepressor Complex Inhibits the JNK Pathway through the Integral Subunit GPS2..

[34] Dhillon AS, Hagan S, Rath O, Kolch W (2007). MAP kinase signalling pathways in cancer.. Oncogene.

[35] Chen W, Houldsworth J, Olshen A, Nanjangud G, Chaganti S (2006). Array comparative genomic hybridization reveals genomic copy number changes associated with outcome in diffuse large B-cell lymphomas.. Blood.

[36] Mullighan CG, Downing JR (2009). Genome-wide profiling of genetic alterations in acute lymphoblastic leukemia: recent insights and future directions.. Leukemia.

